# Impact of Fatty-Acid Labeling of *Bacillus subtilis* Membranes on the Cellular Lipidome and Proteome

**DOI:** 10.3389/fmicb.2020.00914

**Published:** 2020-05-15

**Authors:** Jonathan D. Nickels, Suresh Poudel, Sneha Chatterjee, Abigail Farmer, Destini Cordner, Shawn R. Campagna, Richard J. Giannone, Robert L. Hettich, Dean A. A. Myles, Robert F. Standaert, John Katsaras, James G. Elkins

**Affiliations:** ^1^Department of Chemical and Environmental Engineering, University of Cincinnati, Cincinnati, OH, United States; ^2^Biosciences Division, Oak Ridge National Laboratory, Oak Ridge, TN, United States; ^3^Department of Chemistry, The University of Tennessee, Knoxville, Knoxville, TN, United States; ^4^Biological and Small Molecule Mass Spectrometry Core, The University of Tennessee, Knoxville, Knoxville, TN, United States; ^5^Chemical Sciences Division, Oak Ridge National Laboratory, Oak Ridge, TN, United States; ^6^Neutron Scattering Division, Oak Ridge National Laboratory, Oak Ridge, TN, United States; ^7^Department of Chemistry, East Tennessee State University, Johnson City, TN, United States; ^8^Shull Wollan Center – a Joint Institute for Neutron Sciences, Oak Ridge National Laboratory, Oak Ridge, TN, United States; ^9^Department of Physics and Astronomy, The University of Tennessee, Knoxville, Knoxville, TN, United States; ^10^Department of Microbiology, The University of Tennessee, Knoxville, Knoxville, TN, United States

**Keywords:** *Bacillus subtilis*, biomembranes, proteomics, lipidomics, fatty-acids

## Abstract

Developing cultivation methods that yield chemically and isotopically defined fatty acid (FA) compositions within bacterial cytoplasmic membranes establishes an *in vivo* experimental platform to study membrane biophysics and cell membrane regulation using novel approaches. Yet before fully realizing the potential of this method, it is prudent to understand the systemic changes in cells induced by the labeling procedure itself. In this work, analysis of cellular membrane compositions was paired with proteomics to assess how the proteome changes in response to the directed incorporation of exogenous FAs into the membrane of *Bacillus subtilis*. Key findings from this analysis include an alteration in lipid headgroup distribution, with an increase in phosphatidylglycerol lipids and decrease in phosphatidylethanolamine lipids, possibly providing a fluidizing effect on the cell membrane in response to the induced change in membrane composition. Changes in the abundance of enzymes involved in FA biosynthesis and degradation are observed; along with changes in abundance of cell wall enzymes and isoprenoid lipid production. The observed changes may influence membrane organization, and indeed the well-known lipid raft-associated protein flotillin was found to be substantially down-regulated in the labeled cells – as was the actin-like protein MreB. Taken as a whole, this study provides a greater depth of understanding for this important cell membrane experimental platform and presents a number of new connections to be explored in regard to modulating cell membrane FA composition and its effects on lipid headgroup and raft/cytoskeletal associated proteins.

## Introduction

Biological membranes fulfill many critical roles in the cell; the details of which are rooted in its structure, composition, biochemistry, and biophysical properties. A vivid picture of the cell membrane has emerged from decades of *in vitro* studies of model membranes and a limited number of *in vivo* studies. Perhaps foremost among current questions of the cell membrane center around the existence, composition, size and roles of lipid rafts. The lipid raft hypothesis (Simons and Ikonen, 1997) invokes lateral organization of membrane lipids and proteins into distinct domains in the plane of the membrane to facilitate the assembly and regulation of multi-molecular complexes. This hypothesis provides a compelling rationale for numerous observations relating to membrane trafficking, endocytosis, signal transduction, and other processes (Simons and Toomre, 2000; Simons and Ehehalt, 2002; Shaw, 2006; Allen et al., 2007). It has also been suggested that rafts may contribute to the short-term physical stability of the cell membrane by buffering the mechanical properties of the membrane against rapid changes in temperature, local solvents, and other environmental perturbations (Nickels et al., 2019). Experimental evidence describing lipid rafts has been largely inferential due to the limitations of traditional techniques, and it is widely believed that these features are nanoscopic, as well as transient (Mukherjee and Maxfield, 2004; Lingwood and Simons, 2010), making them difficult to detect *in vivo*.

These difficulties have inspired researchers to turn to techniques such as neutron scattering to resolve the lateral organization of the cell membrane. Neutron scattering has yielded detailed descriptions of lateral organization in model membranes (Nickels et al., 2015), as well as direct evidence of lateral heterogeneity of fatty acids (FAs) within the cell membrane of living cells of *Bacillus subtilis* (Nickels et al., 2017b). Performing scattering experiments on live cells is not trivial however, with contributions to the scattering from the solvent and all biomolecules comprising the cell. Isolating the scattering from the cell membrane required extensive deuteration efforts whereby the isotopic makeup of the biomolecules within the cell are systematically altered to manipulate the scattering length density while introducing a chemically and isotopically defined FA composition to the cell’s membrane. This was achieved via three main strategies in the prior work by Nickels et al. (2017b), the first being cultivation of the cells in partially deuterated growth media conditions to control the background deuterium content of the cellular biomolecules. Next, *de novo* FA synthesis was blocked chemically using cerulenin, an irreversible inhibitor of FabF, or β-ketoacyl-ACP synthase (Wille et al., 1975; Price et al., 2001). Finally, the potential catabolism of the exogenous FAs was prevented by using a Δ*yusL* strain of *B. subtilis*, eliminating a critical enzyme in β-oxidation, FadN, or enoyl-CoA hydratase (Matsuoka et al., 2007).

With FA catabolism and anabolism blocked, Nickels et al. (2017b) used a combination of two FAs – palmitic acid (*normal*-hexadecanoic acid, *n*16:0) and 12-methyltetradecanoic acid (*anteiso*-pentadecanoic acid, *a*15:0) – to rescue cellular growth in the presence of inhibitory concentrations of cerulenin. These FAs constitute a minimal set of one high-melting point (*n*16:0) and one low-melting (*a*15:0) FA sufficient to rescue cell growth, enabling the cell to regulate the fluidity and structure of its membrane. The ability to introduce exogenous FAs enables both chemical and isotopic control of the cell membrane FA content, thus enabling direct observations of the cell membrane thickness and laterally heterogeneous distribution of FAs in the cell membrane using neutron scattering methods (Nickels et al., 2017b). However, it remains unknown what additional changes the labeling method induces, specifically with regard to cell membrane-associated features. In the original study (Nickels et al., 2017b), isotopic labeling of cell membranes was performed using deuterated/hydrogenated fatty acid species. Clearly a more generic labeling is possible using this approach, including additional and unnatural fatty acids, along with a range of other isotopic variants. Here, we have opted to utilize only the standard hydrogenated species replicating the *n*16:0/*a*15:0 combination of the original work since the inclusion of expensive isotopic variants is not the focus of this study, but rather changes in the cell induced by the labeling strategy. Note that the resulting cell membrane contains only the FAs added to the culture (*n*16:0 and *a*15:0), which represents two of the seven native FAs produced in *B. subtilis*. Along with this reduction in compositional diversity, there is an increase in the relative content of *n*16:0 – the high melting temperature component. Recent simulations indicate that increasing amounts of *n*16:0 within a *B. subtilis* lipid extract can induce phase changes at around 20% *n*16:0 content (Mostofian et al., 2019), raising the question of compositionally induced phase separation. It is also expected that there will be changes in the expression of cellular proteins in response to the presence of cerulenin, and likewise a change in the available FAs. It has been reported that cerulenin, a fungal toxin, induces an 8-fold increase in the expression of the target enzyme FabF and it is anticipated that additional differences in expression will be observed due to both an increase in free FA availability as well as a stress response imparted by exposure to the fungal toxin (Schujman et al., 2001). Of particular interest are changes in the expression of lipid raft-associated scaffold proteins such as flotillin (Bickel et al., 1997) and the presence of isoprenoid lipid species and their associated biosynthetic enzymes. Evidence is emerging which suggests a role of isoprenoid species in the structure of lipid rafts, analogous to that of sterols in eukaryotic cells (Sáenz et al., 2012). Indeed, recent work has provided direct evidence that raft disruption by inhibiting isoprenoid lipid synthesis substantially alters the function of dimeric proteins in the microbial cell membrane (García-Fernández et al., 2017). In an effort to better characterize the experimental platform described above, this report provides the results of lipidomic and proteomic analyses detailing the changes induced by the use of cerulenin and exogenous FAs to control the FA composition of the *B. subtilis* cell membrane.

## Results and Discussion

**FIGURE 1:**
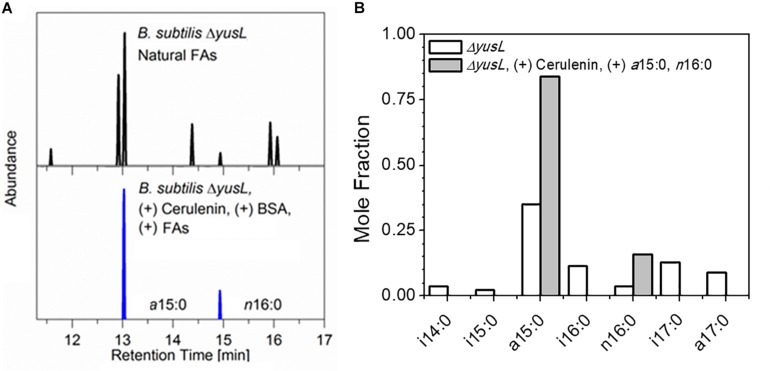
**(A)** *B. subtilis* typically contains a mixture of seven linear and branched saturated FAs. Shown here are GC/MS chromatograms comparing cerulenin-treated Δ*yusL* cells rescued by the addition of two FAs, a15:0 and n16:0 (upper panel) with untreated Δ*yusL* cells (lower panel). **(B)** The distribution is summarized via integration and normalization of the chromatograms. The cerulenin-treated cells incorporate only the two exogenously provided FAs in their membranes, demonstrating the specificity and effectiveness of the labeling strategy.

The control of FA incorporation in *B. subtilis* using the cerulenin method was tested by GC/MS analysis of FA methyl esters (FAMEs) extracted from two aliquots of *B. subtilis* cells containing a deletion of the *fadN* gene which were cultured in a minimal M9 glucose medium prepared with H_2_O (Figure 1). As expected, roughly 90% of the FA composition measured in unlabeled/unfed cells are represented by a distribution of saturated branched (*iso*- and *anteiso-*) FAs (*i*14:0, *a*15:0, *i*15:0, *i*16:0, *i*17:0, *a*17:0), in addition to a small amount of saturated unbranched (*normal-*) FAs (*n*16:0). This is in contrast to the observation of only *a*15:0 and *n*16:0 in the cells treated with cerulenin and supplemented with exogenous *a*15:0 and *n*16:0 FAs. The degree of labeling observed here in hydrogen containing media is in complete agreement with the prior study (Nickels et al., 2017b) where the cells took up the exogenous mixture in an ∼3.5:1 ratio, *a*15:0 to *n*16:0 in a deuterium rich growth media.

Cerulenin exposure has been previously reported to increase the amount of FabF, the enzyme targeted by cerulenin, up to 8-fold (Schujman et al., 2001). As expanded upon below, this regulation is in expected to be associated with an accumulation of FA precursors, yet it is unclear how subsequent rescue of cell growth with exogenous FAs will impact FabF expression and other enzymes within the FA biosynthesis pathway in response to accumulation of acyl-ACP and free FAs due to exogenous addition. Moreover, the introduction of exogenous FAs may also impact the reverse process of FA degradation. With the deletion of the *fadN* gene, the addition of free FAs might result in accumulating *trans*-2-enoyl-CoA and 3-hydroxyacyl-CoA products. Thus, attention was first directed toward understanding the changes in the relative levels of the enzymes involved in these processes.

**FIGURE 2:**
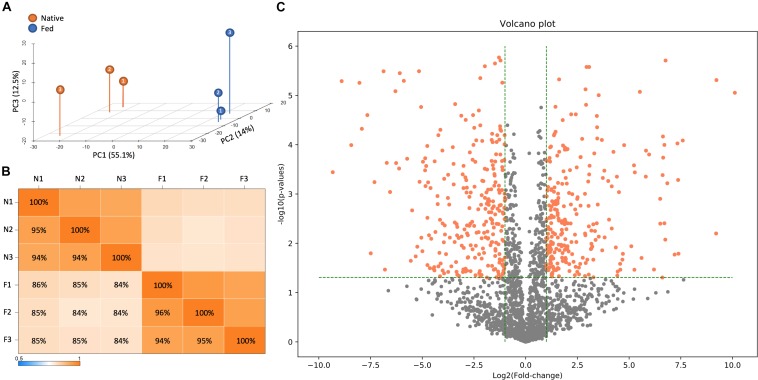
LC-MS/MS-based proteomic measurements revealed differences in protein content between *B. subtilis* cells harboring the *fadN* (*yusL*) deletion and exposed to cerulenin and exogenous FAs (F) and cells not exposed to cerulenin and exogenous FAs (N). **(A)** Principle component analysis illustrates broad differences in protein content between these conditions which are further summarized in a correlation matrix **(B)**. **(C)** A volcano plot is useful to visually summarize the number of proteins which display statistically significant (>0.05 on the y-axis) changes in the protein abundance (greater than an absolute value of 1.0 on the x-axis).

To accomplish this task, LC-MS/MS-based proteomic measurements were performed for *B. subtilis* cells harboring the *fadN* (*yusL*) deletion that have been allowed to synthesize the natural complement of FAs and compared to cells treated with cerulenin and then fed *n*16:0 and *a*15:0 FAs to rescue growth. Cells were harvested at mid-log phase and proteomes compared across both conditions (*n* = 3). Top-level proteome QC analysis indicates that sample replicates grouped accordingly and were largely segregated across principle component 1, which represents the two feeding conditions (Figure 2A). In total, 2026 *B. subtilis* proteins were quantified and statistically assessed by Student’s *T*-test (Supplementary Table S1; significance requires *p* ≤ 0.05 and fold-change ≥ 1 log2 units). Of these proteins, 511 exhibited significant differences in abundance whereby 253 and 258 were either up- or down-regulated upon cerulenin treatment and rescuing growth via the defined exogenous FAs, respectively (Figures 2B,C). FadN was absent from both sample groups as expected. These differentially abundant proteins were then further analyzed to elucidate the systemic impact of feeding *n*16:0 and *a*15:0 FAs to *B. subtilis* with impaired FA metabolism via cerulenin treatment (FabF inhibition) and deletion of *fadN.*

**FIGURE 3:**
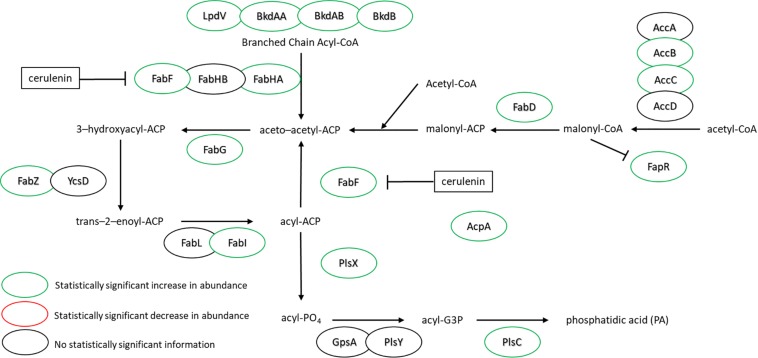
FA synthesis pathway of *B. subtilis* illustrating the observed changes in protein expression in response to both cerulenin addition and rescue of cellular growth with exogenous FA supplementation using *n*16:0 and *a*15:0 FAs. Cerulenin irreversibly binds to FabF, blocking FA synthesis. The observed 6.6-fold increase in FabF is consistent with previous reports (Schujman et al., 2001). These results also indicate that many other proteins in the pathway are overexpressed in response to these conditions, including the regulation protein, FapR. For locus tag, UniProt Description, log2 change in expression, and *p*-values for each differentially expressed protein (see Supplementary Table S2).

Figure 3 highlights the changes in abundance for a number of enzymes within the FA biosynthesis pathway – all showing either increases or no change at all upon cerulenin treatment and rescue with *n*16:0 and *a*15:0 FAs. The target of cerulenin, FabF, was ∼6.5-fold more abundant which is consistent with the prior report (Schujman et al., 2001). Beyond this, many other components of the pathway were also found in higher abundance in the presence of cerulenin and exogenous FAs. These enzymes are all controlled via the *fap* regulon, suggesting that co-regulation is the mechanism. The regulation of the FA biosynthesis pathway occurs via the protein FapR (Schujman et al., 2003) which binds to DNA, preventing transcription of these enzymes and resulting in autoregulation. Malonyl-CoA, a precursor molecule, binds to FapR, releasing it from its DNA binding site and enabling transcription of these enzymes. The synthesis of acetyl-CoA also seems to be impacted, with the enzyme AcsA, acetyl-CoA synthetase, exhibiting a large increase in abundance (∼4-fold). The increased expression of the *fap* regulon and FapR itself suggests that a large amount of malonyl-CoA has accumulated due to the blockage of FabF. Additionally, we observe a statistically significant 3-fold increase in the expression of the polyketide synthase, PksM, which may be in response to accumulation of acetyl-CoA or malonyl-CoA (Supplementary Table S1).

**FIGURE 4:**
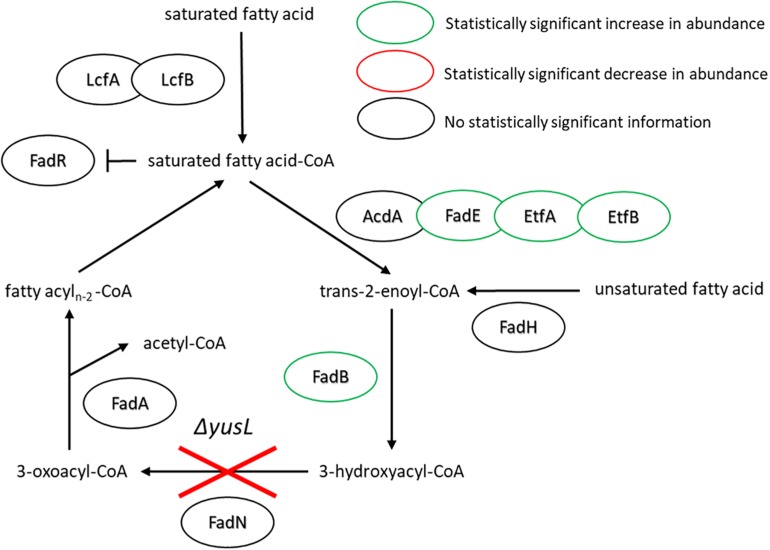
FA degradation pathway of *B. subtilis* illustrating the observed changes in protein expression in response to both cerulenin addition and rescue of cellular growth with exogenous FA supplementation (*n*16:0 and *a*15:0). The Δ*yusL* deletion has removed FadN in both cases, yet there appears to be an increase in the amount of enzymes preceding the deletion when the cells have been subjected to the FA labeling strategy. For locus tag, UniProt Description, log2 change in expression, and *p*-values for each differentially expressed protein (see Supplementary Table S2).

With regard to the FA degradation pathway, there are fewer proteins represented/identified relative to FA synthesis. However, FadE, EtfA, EtfB, and FadB have all increased in abundance (1.5, 10.8, 2.3, and 3.9, respectively) (Figure 4). Interestingly, the observed increases all appear in proteins encoded upstream of the *fadN* deletion. It would be reasonable to assume that the uptake of exogenous FAs will lead to accumulation of corresponding metabolites. Global regulation of the FA degradation pathway is controlled by FadR, which binds DNA, blocking transcription. Long chain FAs bind to FadR and release it from its operator sites, enabling production of these FA degradation enzymes (Matsuoka et al., 2007).

The increase in relative *n*16:0 content with respect to the native membrane composition is a potentially important consideration, as *n*16:0 is the natural FA with the highest melting temperature. This may imply that the labeled cell membranes contain more ordered acyl regions, with recent simulations demonstrating precisely this effect (Mostofian et al., 2019). There, it was shown that an *n*16:0 content of ∼20% is sufficient to induce a phase change in a model of the *B. subtilis* cell membrane. Cells are known to actively regulate the fluidity of the cell membrane however (Zhang and Rock, 2008), posing the question of what other differences might be occurring to counter this. Therefore, we used LC-MS lipidomics to interrogate other changes in the lipid compositions. The composition of the phospholipid headgroups affects the fluidity of the membrane along with the length of the FA chain.

**FIGURE 5:**
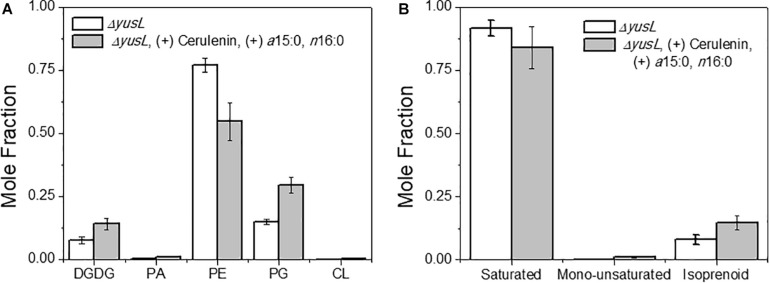
**(A)** The distribution of lipids was altered due to the isotopic labeling and FA restriction. Lipids are organized by headgroup and the normalized mole fraction plotted. A clear decrease in PE, and corresponding increase in PG is observed. Other species also exhibit smaller changes, such as the diacylglycerol, PA, and CL content all increasing. **(B)** A broader look at the hydrophobic lipid components compliments the above analysis of saturated FAs. Here we see that there are also changes in the content of mono-unsaturated and isoprenoid species.

**FIGURE 6:**
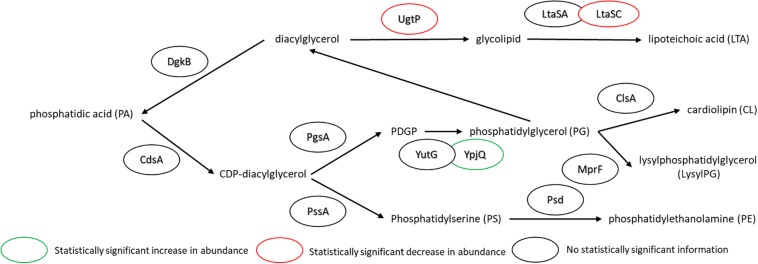
Pathway for lipid biosynthesis, annotated with statistically significant changes in protein expression upon the use of cerulenin and exogenous FAs to label the cell membrane. An increase in the production of PG is observed (see Figure 4), which may be associated with an increase in YpjQ (Sierro et al., 2007) involved in producing PG lipids and decreases in both UgtP and LtaSC which utilize PG lipids to generate lipoteichoic acid (Percy and Gründling, 2014). For locus tag, UniProt Description, log2 change in expression, and *p*-values for each differentially expressed protein (see Supplementary Table S2).

The major changes observed in the cell membrane composition using this method were: a decrease in phosphatidylethanolamine (PE) headgroup content and corresponding increase in phosphatidylglycerol (PG) lipids (Figure 5A), an increase in isoprenoid lipid content from ∼8 to ∼15%, and a small increase in mono-unsaturated lipid content associated with an induced stress response (Figure 5B). This is in addition to the changes in the saturated FA composition detailed above. The lipid headgroups can also have a substantial impact upon cell membrane fluidity. A comparison of melting temperatures for lipids with PE vs. PG headgroups, but the same FAs illustrates this phenomenon [1,2-dipalmitoyl-sn-glycero-3-phosphoethanolamine (DPPE; PE32:0) has a Tm of 63°C vs. 1,2-dipalmitoyl-sn-glycero-3-phosphoglycerol (DPPG; PG32:0) which has a Tm of 41°C] (Tm tabulated at avantipolarlipids.com). The native cell membrane of *B. subtilis* 168 contains PE, PG, cardiolipin (CL), and lysyl-PG lipids, in addition to ∼30 percent neutral lipids which are mostly diacylglycerol (Bishop et al., 1967; Den Kamp et al., 1969; Clejan et al., 1986; Kawai et al., 2004; Nickels et al., 2017a; Figure 5A). In the labeled cells, the substantial decrease in PE, and corresponding increase in PG, would have a fluidizing effect upon the membrane, potentially counteracting the ordering effect of increasing *n*16:0 FA content. The enzymes associated with production of lipid headgroups show a series of changes which might account for the accumulation of PG (Figure 6). The observed increase in YpjQ abundance (3.4-fold) would be associated with an increase in the production of PG (Sierro et al., 2007) while decreases in both UgtP and LtaSC would reduce flux to lipoteichoic acid from PG (Percy and Gründling, 2014). This increase of production and decrease in utilization may explain the observed accumulation of PG lipids when the cerulenin and FA labeling method is applied. No statistically significant change in expression was obtained regarding the enzymes responsible for PE synthesis, PssA, or Psd.

Other expected changes that result from decreased membrane fluidity induced here include unsaturation of membrane lipids (Aguilar et al., 1998) and an increase in the amount of anteiso-branched-chain FA – the latter of which is controlled via an isoleucine dependent mechanism (Klein et al., 1999). Here, the branched chain response is both blocked in the FA biosynthesis pathway and the culture supplemented with BSA as a carrier for the exogenous FAs, the latter of which also provides isoleucine to the cell. Isoleucine concentration is also an important part of the FA desaturation mechanism (Cybulski et al., 2002). Here, we observe a very small amount (∼1%) of FA mono-unsaturation during FA restriction in *B. subtilis*. The associated protein, a FA desaturase, Des, is reported to be a Δ*5* desaturase in *B. subtilis*. The protein and the associated desaturation events are connected to thermosensing and hence the homeoviscous adaptation of the cell membrane through the sensor kinase DesK and the response regulator DesR (Aguilar et al., 2001). However, no statistically significant changes in the content of this protein were observed. One possible explanation for this lack of response is the reported sensitivity to isoleucine concentration (Cybulski et al., 2002) which again may be suppressed with the supplementation of the growth media with bovine serum albumin (BSA) which is added as a FA carrier to increase the bioavailibilty of insoluble FAs in culture, which contains isoleucine.

Numerous changes were observed in the abundance of enzymes associated with isoleucine metabolism. The abundance of enzymes associated with the synthesis of isoleucine were predominantly lowered; IlvA, IlvB, IlvC, and IlvE were all observed at lower amounts (1.6-,1. 8-,1. 6-, and 31.8-fold lower, respectively) when the cerulenin/FA labeling approach was applied. One exception is IlvD, which showed a small increase of 1.8-fold in expression (Supplementary Table S1). Many of these enzymes are shared with those in leucine synthesis, and the leucine specific synthesis enzymes LeuA, LeuB, LeuC, and LeuD were all measured at lower amounts as well (7.5-,3.9-, 3.7-, and 6.6-fold lower, respectively). On the other hand, an increase in abundance was observed for (iso)leucine degradation enzymes Bcd, BkdAA, BkdAB, BkdB, LpdV, Ptb, and Buk (10.9-,5.6-,2.8-,4.1-,8.3-,165. 3-, and 5.3-fold, respectively). Interestingly, there were no statistically significant differences observed in the proteins controlling the global regulation of isoleucine metabolism, specifically CcpA, CodY, or TnrA (Tojo et al., 2005). Finally, an increase in the membrane transport protein for isoleucine, BcaP, of 1.6 times was observed. Taken together, this suggests a cell population reducing synthesis of isoleucine but increasing the amount of a membrane transporter for isoleucine, all whilst increasing the number of enzymes used for isoleucine degradation. This may support the notion of a blocked stress response due to the labeling strategy.

**FIGURE 7:**
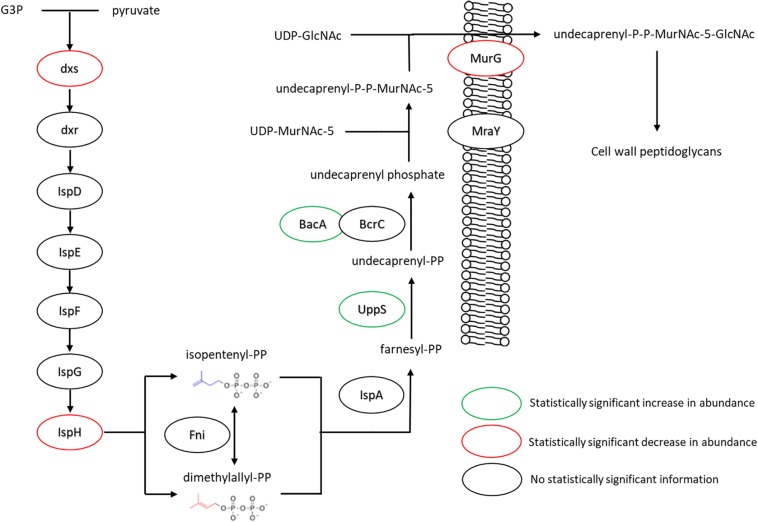
Changes in expression levels of enzymes involved in the production of isoprenoid lipids and their use in peptidoglycan export. Enzymes involved in generating precursors to isoprenoid lipids appeared to be down regulated, while those involved in extending farnesyl diphosphate to undecaprenyl phosphate were seen in increased quantity. The transferase MurG, involved in peptidoglycan export, was seen to be expressed in lower amounts. For locus tag, UniProt Description, log2 change in expression, and *p*-values for each differentially expressed protein (see Supplementary Table S2) (UDP, Uridine diphosphate; MurNAc5, N-acetylmuramic acid pentapeptide; GlcNAc, N-acetylglucosamine).

The changes in isoprenoid lipid content were more substantial, increasing from ∼8 to ∼15% (Figure 5B). Isoprenoid lipids were not analyzed in our previous work (Nickels et al., 2017b). However, the LC/MS method used for this study allows these molecules to be detected and their relative abundance can be determined. We defined isoprenoid lipids as species from the LC/MS data with three or more unsaturations, including farnesyl-, geranyl-, and other prenyl-molecules. We observe statistically significant changes in proteins of the isoprenoid pathway (Hess et al., 2013), specifically; Dxs, IspH, and UppS. These changes are illustrated in Figure 7, showing lower abundance of enzymes within the MEP pathway, which is responsible for the production of isoprenoids in *B. subtilis* (Campbell and Brown, 2002; Laupitz et al., 2004; Takagi et al., 2004; Julsing et al., 2007). The largest of these isoprenoids, undecaprenyl-PP, is an isoprenoid lipid molecule used in the transfer of sugars to the cell wall from the cytoplasmic space; this pathway is also included in Figure 7. Some of the enzymes associated with processing farnesyl diphosphate to undecaprenyl phosphate were upregulated (BacA 2.4-fold and UppS 1.7-fold), possibly explaining the increased amount of isoprenoid lipid observed. However, the enzyme used to transport the molecules to the cell wall, undecaprenyl-PP-MurNAc-pentapeptide-UDPGlcNAc GlcNAc transferase (MurG) was about 8-fold lower in abundance for the fed condition.

The increase in isoprenoid lipid content invite a consideration of how it might be connected to lateral organization within the cell membrane. Lipid domains, or rafts, are a key organizational feature of the cell membrane (Simons and Ikonen, 1997; Brown and London, 1998). Indeed, the observation of rafts in the *B. subtilis* cell membrane was the motivation for this labeling protocol (Nickels et al., 2017b). It is also thought that some isoprenoid lipids, like carotenoids and hopanoids, function analogously to cholesterol and sphingolipids in eukaryotic organisms (Welander et al., 2009; Doughty et al., 2014; Sáenz et al., 2015). A recent review of functional microdomains in *B. subtilis* (Wagner et al., 2017) highlights the potential role of isoprenoid lipids. Though the specific isoprenoid molecules are not yet known, there is genetic evidence (López and Kolter, 2010; Feng et al., 2014) that they are synthesized perhaps via YisP, which is thought to be a farnesyl diphosphatase. This potentially identifies farnesol as a component of microdomains in *B. subtilis* membranes. However, we did not detect statistically significant changes in this protein and our lipidomic results indicate a more diverse set of isoprenoid molecules being generated. Evidence has also emerged for lipid domains rich in PE (Vanounou et al., 2003; Nishibori et al., 2005), PG (Vanounou et al., 2003; Barák et al., 2008), and cardiolipin (Kawai et al., 2004) in *B. subtilis*. These reports center on spiral like features in the long aspect of the cell, apparent when using specific lipid soluble fluorescent dyes, as well as cardiolipin and/or PE in enriched regions at the cell poles and in the septal region of the cell membrane.

It is known that *yisP* is a part of the SigB regulon. The SigB regulon refers to a set of over 200 genes broadly associated with bacterial-fungal interactions. It is logical then, when using a fungal toxin such as cerulenin, to expect SigB to have a role in the cellular response. Our proteomic data shows that indeed a large number of these proteins for which we can make a statistically significant comparison are expressed at a higher amount, while the sigma factor itself, SigB, appears to be present at about seven times less than is seen without cerulenin and FAs present. The mechanism of SigB activation is thought to be based on repression by an anti-sigma factor, RsbW, and its antagonist RsbV (Delumeau et al., 2002). Both regulator molecules are increased by ∼2-fold.

Another sigma factor, SigW, also appears to be strongly impacted in response to cerulenin/FA conditions. SigW, a factor associated directly to membrane stress (Helmann, 2016), is at least 45 times less abundant upon application of cerulenin/FA supplementation. In addition, the proteins within the SigW regulon appear to be lower as well. This includes the lipid raft associated protein flotillin, which is discussed below. The only protein within the SigW regulon exhibiting increased abundance are the FA synthesis enzymes FabHA and the target of cerulenin, FabF. Clearly these proteins are also regulated by FabR which is counteracting the suppression of other SigW-regulated proteins.

These organizational features of the cell membrane lipids are connected to the cell wall and cytoskeleton (Muchová et al., 2011). As mentioned above, flotillin is one clear example of a lipid raft associated protein (López and Kolter, 2010; Bach and Bramkamp, 2013). Indeed, FloT, also known as YuaG, is equivalently seen to be associated with bacterial microdomains (Donovan and Bramkamp, 2009; López and Kolter, 2010); and, FloT possesses 39% sequence identity to Flotilin-1 found in eukaryotic cells (Langhorst et al., 2005; López and Kolter, 2010). In this study, flotillin abundance was measured at approximately five-time lower upon cerulenin/FA exposure. Several studies have built upon these observations and investigated the role of FloT in microbial membranes where FloT abundance was closely associated with the efficacy of raft associated antibiotic resistance enzymes (Schneider et al., 2015; Dempwolff et al., 2016). Another cytoskeletal protein associated with membrane organization, MreB, exhibited 1.4-fold decreased abundance. This actin-like cytoskeletal protein is associated with MurG (Divakaruni et al., 2007; Mohammadi et al., 2007; White et al., 2010), which as shown in Figure 7, is directly involved in cell wall peptidoglycan export – specifically by binding with and flipping an isoprenoid lipid. MurG is thought to bind preferentially to cardiolipin (Boots et al., 2003) and was seen to have decreased abundance similar to MreB.

## Conclusion

The engineering of *B*. *subtilis* membrane components, as detailed here, provides an opportunity for *in vivo* membrane biophysical measurements using chemically and isotopically defined cell membranes. Though this can be accomplished by conditionally blocking *de novo* FA synthesis via addition of cerulenin and rescuing cellular growth by providing an exogenous supply of FAs, the systemic effects of these treatments remained unknown. The LC/MS analysis employed here elucidates the changes brought upon by this labeling strategy, most notably in the lipid headgroup composition and isoprenoid lipid content as well as the proteomic effects that underpin these specific metabolic pathways.

After demonstrating the uptake of the desired FA composition, the lipidomic analysis has revealed an alteration in lipid headgroup distribution, with an increase in phophatidylglycerol lipids and decrease in phosphatidylethanolamine lipids, possibly providing a fluidizing effect on the cell membrane. Increases in the abundance of enzymes in the FA biosynthesis and degradation pathways are also observed, consistent with earlier reports of cellular response to cerulenin exposure. The inhibition of FA biosynthesis and subsequent FA supplementation appears to frustrate some of the expected cellular responses to decreased bilayer fluidity, such as an increase in anteiso FA content and desaturase activity. Changes to cell wall enzymes and isoprenoid lipid production are also observed, with the content of isoprenoid lipids found to have increased. This may serve to influence membrane organization. Indeed, the well-known lipid raft associated protein flotillin was found to be substantially less abundant in the labeled cells. Though other changes to the proteome upon cerulenin treatment/FA rescue have been observed, and are summarized in the Supporting Information, this report has focused upon the membrane-related findings to provide specific context for future membrane labeling experiments using this platform. Among such future efforts, the use of a more representative natural set of supplemental fatty acids would be an important control, in addition to alternative strategies to accomplish suppression of fatty acid synthesis/degradation, such as CRISPRi/dCas9 approaches, which may or may not be more limited in their action.

This work also provides another data point in the emerging picture of functional membrane domains and lateral organization within microbial cell membranes. Specifically, it is shown that controlling cell membrane composition induces changes in both cytoskeletal proteins, MurG and MreB, and with proteins thought to be lipid raft associated such as FloT. Given that the key function of lipid rafts is to organize protein “cargo” within the plane of the cell membrane, the induced changes in the overall amount of these proteins in response to FA composition is intriguing. The FAs taken up by the cell might be expected to generate a less fluid bilayer, compensated by the described changes in lipid headgroups and isoprenoid lipid content. However, those responses are occurring in classes of molecules demonstrated to, or suspected of, organizing the cell membrane laterally. Disruption or alteration of that organization will have inevitable effects on cellular function, which this data may suggest. Taken together, this study provides a greater depth of understanding for this important cell membrane experimental platform and argues for careful analysis of numerous dimensions of cell membrane and cellular proteome modulation via this or similar FA labeling strategies.

## Materials and Methods

### Microbial Sample Preparation

Cell suspensions of *Bacillus subtilis* 168 were prepared essentially as previously described (Nickels et al., 2017b). Strain BKE32840 (Δ*yusL*) was obtained from the Bacillus Genetic Stock Center (The Ohio State University, Columbus, OH, United States). The culture medium used for producing samples was M9 minimal medium containing 0.4% (*w/v*) glucose and supplemented with 50 mg/L of L-tryptophan (Harwood and Cutting, 1990). Solid media were prepared by the addition of 1.5% Noble Agar (Difco). Erythromycin was added to 0.5 μg/mL for routine maintenance of BKE32840. Broth cultures were incubated at 37°C with shaking at 250 rpm. FA feeding experiments included cultures supplemented with 8 mg/L each of *a*15:0 and *n*16:0 from 25 mg/mL stock solutions in ethanol, along with 10 g/L of FA-free BSA. Cerulenin (Alfa Aesar) was added to a final concentration of 50 μg/mL. Non-fed cultures were grown in M9 minimal medium with 10 g/L BSA but without FAs or Cerulenin. Cells were harvested at mid-log phase (∼0.8 OD_600_) by centrifugation at 6,000 × *g* for 15 min and washed three times in sterile phosphate buffered saline (pH 7.4).

### Saturated Lipid Extraction From *B. subtilis* and Fatty Acid Methyl Ester Analysis (FAME)

Total lipid extraction was performed on whole cells using a modified Bligh and Dyer method (Bligh and Dyer, 1959; Lewis et al., 2000). Briefly, cells were pelleted by centrifugation at 6,000 × *g* for 15 min, followed by three washes in 1% (*w/v*) NaCl. Lyophilized cell pellets were stored at −80°C until needed. The lyophilized cell pellets were crushed into a coarse powder and transferred to a separatory funnel. For every 10 mg of cell mass, solvents were added as follows: 3 mL of chloroform, 6 mL of methanol, and 2.4 mL of water were added, in that order, with vortexing after each addition. This mixture formed a single phase and was held for 18 h at room temperature with occasional agitation. After 18 h, 3 mL of chloroform and 3 mL of water were added to induce phase separation. Lipids were recovered from the lower chloroform phase by rotary evaporation of the solvent and used to prepare FAMEs for analysis.

FAME samples were prepared by acidic methanolysis of lipid extracts (Ichihara and Fukubayashi, 2010). Each sample extract was transferred to a 10 mL screw top test tubes and dried under a stream of argon gas, prior to the addition of 1 mL of concentrated HCl/methanol (10% *v/v*). The test tube was purged with Ar and sealed, then heated to 85°C for 2 h. After allowing the tube to cool, 1 mL of water and 1 mL of hexane were added, mixed, then allowed to form two separate phases. The upper hexane phase containing FAMEs (∼700 μL) was transferred to a 2 mL autosampler vial and loaded in an Agilent 7692A autosampler for subsequent analysis of FA content by gas chromatography/mass spectrometry (GC/MS) using an Agilent 5890A gas chromatograph with a 5975C mass-sensitive detector operating in electron-impact mode (Agilent Technologies, Santa Clara, CA, United States). For detailed GC/MS methods, see Nickels et al. (2017b). ChemStation Enhanced Data Analysis software (Agilent Technologies, Santa Clara, CA, United States) and the NIST mass spectra library (revision 2011) were used to assign and quantify the chromatogram peaks and mass spectra.

### LC-MS/MS-Based Proteomic Analysis of Treated and Untreated *B. subtilis* Cells

Whole cell lysates were prepared by bead beating *B. subtilis* cell pellets (∼50 mg each) in 300 μL of 4% sodium deoxycholate (SDC), 5 mM DTT, 100 mM ammonium bicarbonate (ABC) pH 8.0 using 0.15 mM zirconium oxide beads. The crude lysates were then heat-treated at 95°C for 10 min and centrifuged at 21,000 × g for 10 min to defoam and remove debris. Cleared lysates (250 μL) were then transferred to 10 kDa MWCO spin columns (Vivaspin 500; Sartorius) and cysteines were blocked with 15 mM iodoacetamide for 30 min at room temperature in dark. Samples were then centrifuged at 12,000 × g to remove initial lysis buffer. Proteins trapped atop the filter were then washed with 500 μL ABC buffer and the flow through discarded. Washed proteins were then resuspended in 500 μL of ABC buffer and the concentration was measured using a bicinchoninic acid assay (Pierce^TM^ BCA Protein Assay Kit, Thermo Scientific^TM^). Proteins were digested to peptides with two sequential aliquots of sequencing-grade trypsin (Promega Corp., Madison, WI, United States) at a 1:50 enzyme:protein ratio (*w/w*), initially overnight then followed by 4 h at room temperature. The tryptic peptide solution was then spin-filtered to collect tryptic peptides and adjusted to 1% formic acid to precipitate residual SDC. The precipitated SDC was removed from the peptide solution using water-saturated ethyl acetate. Peptide samples were then concentrated via SpeedVac (Thermo Fisher) and quantified by BCA (Pierce). Peptides were adjusted to a final concentration of 0.5 μg/μL prior to LC-MS/MS analysis.

Peptide samples were analyzed by automated mini-MudPIT LC-MS/MS analysis using an Ultimate 3000 HPLC plumbed directly in-line with a Q Exactive Plus mass spectrometer (Thermo Scientific) outfitted with triphasic MudPIT back column (RP-SCX-RP) coupled to an in-house pulled nanospray emitter packed with 30 cm of 5 μm Kinetex C18 RP resin (Phenomenex). For each sample, 9 μg of peptides were autoloaded, desalted, separated and analyzed across three successive salt cuts of ammonium acetate (35, 100, and 500 mM), each followed by 105 min organic gradient as previously described (Clarkson et al., 2017). Eluting peptides were measured and sequenced by data-dependent acquisition on the Q Exactive.

MS/MS spectra were searched against the *Bacillus subtilis* subsp. subtilis str. 168 (GCF_000009045.1) protein database, concatenated with common contaminant proteins, using the Tide-search algorithm (Diament and Noble, 2011). The following parameters were utilized: parent mass tolerance of 10 ppm, reverse decoy format, a static modification on cysteine (+57.0214 Da), and a dynamic modification to methionine (+15.9949 Da). The rest of the parameters were kept at default settings. Peptide spectrum matches were filtered and rolled up to proteins using Percolator (Käll et al., 2008) employing default parameters. MS1 apex intensities were assigned for all identified peptides using moFF (Argentini et al., 2016). MoFF parameters were as follows: 10 ppm precursor mass tolerance, XIC window = 4 min, and peak apex window = 60 s. Peptide intensities from each salt pulse were summed to their respective proteins per sample. Protein intensities were then normalized by protein length and overall abundance per MS experiment. A minimum of two distinct peptides were required for a protein identification. For quantitative comparisons, only proteins identified in 2 out of 3 replicates were considered. Protein abundance values were log2-transformed and missing values imputed to simulate the mass spectrometer’s limit of detection. Differentially abundant proteins between control and treatment were assessed by Student’s *T*-test (significance = *p* ≤ 0.05 and log2 fold change ≥ 1).

### LC-MS-Based Lipidomic Analysis of Treated and Untreated *B. subtilis* Cells

Lipids were extracted using a protocol modified from that published by Guan et al. (2010). The cell pellet was collected and resuspended in 1 mL of extraction solvent that consisted of 95% EtOH, water, diethyl ether, pyridine, and 4.2 N ammonium hydroxide (to adjust pH) in a 15:15:5:1:0.18 ratio of volumes. Glass beads (100 μL) were added, and the sample was vortexed before being placed into a 60°C water bath for 20 min. At this point, the extraction vessel was centrifuged at 10,000 × g for 10 min. The supernatant was transferred to a separate vial. This extraction procedure was then repeated with the remnants of the cell pellets and glass beads, and the resulting supernatant was combined with the previously collected one. A final extraction of the glass beads was performed using 300 μL of water saturated butanol and 150 μL of water. After mixing the sample with this solution, it was centrifugation at 10,000 × g for 2 min, and the top butanol phase was collected and combined with the other supernatants. The aqueous phase was re-extracted with 300 μL of water saturated butanol, vortexed, and centrifuged at 10,000 × g for 2 min; and the top butanol phase was added to the same vial for drying. Lipid extracts were dried under N_2_, resuspended in 300 μL of MeOH:CHCl_3_ 9:1 and placed in an autosampler vial for MS analysis.

An Ultimate 3000 autosampler and UPLC pump (Thermo Fisher Scientific, San Jose, CA, United States) was used to separate extracted lipids on a Kinetex HILIC column (150 × 2.1 mm, 2.6 μm) (Phenomenex, Torrance, CA, United States). Analytes were introduced to the Exactive Orbitrap mass spectrometer (Thermo Fisher Scientific, San Jose, CA, United States) via an electrospray ionization (ESI) source. The total run time for each analysis was 35 min, and mobile phases A and B consisted of 10 mM aqueous ammonium formate pH 3 in 93% (*v/v*) ACN and 10 mM ammonium formate pH 3, respectively. A flow rate of 0.2 mL/min was used for the gradient elution, and the gradient was as follows: *t* = 0 min 100% A, *t* = 15 min 81%A, *t* = 15.1 min 48% A, *t* = 25 min 48% A, *t* = 25.1 min 100% A, *t* = 35 min 100% A. The temperature of the column oven was 25°C and the temperature of the autosampler was 4°C.

All samples were analyzed in positive and negative mode with a resolution of 140,000 k using a scan range of 100–1,500 *m/z*. The ESI source settings were the same for both positive and negative mode analyses. The heated capillary inlet temperature was set to 350°C, the spray voltage was 4 kV, the sheath gas flow was set to 25 units, and the auxiliary gas was set to 10 units. The standard calibration protocol from Thermo Fisher was performed approximately every 48 h to ensure accurate external mass calibration.

The Maven software package was used to generate extracted ion chromatograms and integrate the resulting chromatographic peaks (Melamud et al., 2010). Lipids were annotated using both the exact *m/z* of the parent ion and the respective retention times. Concentrations of the lipids were calculated using external calibration curves constructed lipid standards (Avanti Polar Lipids, Alabaster, AL, United States) from each of the following lipid classes: sulfoquinosyldiacylglycerol (SQDG), phosphatidylserine (PS), phosphatidylglycerol (PG), phosphatidylethanolamine (PE), phosphatidic acid (PA), cardiolipin (CL), ceramide (Cer).

## Author’s Note

This manuscript has been authored by UT-Battelle, LLC under Contract No. DE-AC05-00OR22725 with the U.S. Department of Energy. The United States Government retains and the publisher, by accepting the article for publication, acknowledges that the United States Government retains a non-exclusive, paid-up, irrevocable, world-wide license to publish or reproduce the published form of this manuscript, or allow others to do so, for United States Government purposes. The Department of Energy will provide public access to these results of federally sponsored research in accordance with the DOE Public Access Plan (http://energy.gov/downloads/doe-public-access-plan).

## Data Availability Statement

All raw mass spectra for the proteome measurements have been deposited into the ProteomeXchange repository with the following accession numbers: (MassIVE Accession: MSV000084229, ProteomeXchange: PXD015150, FTP link to files: ftp://MSV000084229@massive.ucsd.edu).

## Author Contributions

JE, JN, DM, RS, and JK conceived the study and designed the experiments. SC prepared all microbial samples needed for the study. SP and RG performed the proteome measurements. AF, SRC, and JN performed the lipidomics work. JN, DC, SP, RG, AF, RH, and JE analyzed and integrated the data. JN, DC, RG, JK, and JE were primarily responsible for manuscript preparation. All co-authors have inspected and approved the final submitted version.

## Conflict of Interest

The authors declare that the research was conducted in the absence of any commercial or financial relationships that could be construed as a potential conflict of interest.
