# Supplementary material for: Impact of Fatty-Acid Labeling of Bacillus subtilis Membranes on the Cellular Lipidome and Proteome
Source: Front Microbiol. 2020 May 15;11:914. doi: 10.3389/fmicb.2020.00914 (PMC7243436; doi:10.3389/fmicb.2020.00914)
Supplement: Supplementary file 2 [file Table_2.DOCX]

**Supplemental Table S2:** locus tag, UniProt Description, log2 change in expression and p-values for each differentially expressed protein displayed in Figures 3, 4, 6, and 7.

| **Locus** | **UniProt Description** | **Gene Call** | **Log2 Change in Abundance, Fed vs. Unfed Cells** | **p-value** |
| --- | --- | --- | --- | --- |
| **Figure 3** |  |  |  |  |
| CAB14337.2 | Dihydrolipoyl dehydrogenase (EC 1.8.1.4) (Dihydrolipoamide dehydrogenase) (E3 component of branched-chain alpha-keto acid dehydrogenase complex) (LPD-Val) | *lpdV* | 3.06 | 0.0045 |
| CAB14336.1 | 2-oxoisovalerate dehydrogenase subunit alpha (EC 1.2.4.4) (Branched-chain alpha-keto acid dehydrogenase E1 component alpha chain) (BCKDH E1-alpha) | *bkdAA* | 2.49 | 0.0013 |
| CAB14335.1 | 2-oxoisovalerate dehydrogenase subunit beta (EC 1.2.4.4) (Branched-chain alpha-keto acid dehydrogenase E1 component beta chain) (BCKDH E1-beta) | *bkdAB* | 1.47 | 0.0016 |
| CAB14334.1 | Lipoamide acyltransferase component of branched-chain alpha-keto acid dehydrogenase complex (EC 2.3.1.168) (Branched-chain alpha-keto acid dehydrogenase complex component E2) (BCKAD-E2) (BCKADE2) (Dihydrolipoamide acetyltransferase component of branched-chain alpha-keto acid dehydrogenase complex) (Dihydrolipoamide branched chain transacylase) (Dihydrolipoyllysine-residue (2-methylpropanoyl)transferase) | *bkdB* | 2.02 | 0.0001 |
| CAB14880.1 | Acetyl-coenzyme A carboxylase carboxyl transferase subunit alpha (ACCase subunit alpha) (Acetyl-CoA carboxylase carboxyltransferase subunit alpha) (EC 6.4.1.2) | *accA* | -0.05 | 0.4479 |
| CAB14366.1 | Biotin carboxyl carrier protein of acetyl-CoA carboxylase (BCCP) | *accB* | 1.08 | 0.0064 |
| CAB14365.2 | Biotin carboxylase 1 (EC 6.3.4.14) (Acetyl-CoA carboxylase subunit A 1) (ACC 1) (EC 6.4.1.2) | *accC* | 0.90 | 0.0330 |
| CAB14881.2 | Acetyl-coenzyme A carboxylase carboxyl transferase subunit beta (ACCase subunit beta) (Acetyl-CoA carboxylase carboxyltransferase subunit beta) (EC 6.4.1.2) | *accD* | 0.25 | 0.5533 |
| CAB12975.1 | 3-oxoacyl-[acyl-carrier-protein] synthase 2 (EC 2.3.1.179) (3-oxoacyl-[acyl-carrier-protein] synthase II) (Beta-ketoacyl-ACP synthase II) (KAS II) | *fabF* | 2.73 | 0.0001 |
| CAB12857.1 | 3-oxoacyl-[acyl-carrier-protein] synthase 3 protein 2 (EC 2.3.1.180) (3-oxoacyl-[acyl-carrier-protein] synthase III protein 2) (Beta-ketoacyl-ACP synthase III 2) (KAS III 2) (bFabH2) | *fabHB* | 9.21 | 0.0063 |
| CAB12974.1 | 3-oxoacyl-[acyl-carrier-protein] synthase 3 protein 1 (EC 2.3.1.180) (3-oxoacyl-[acyl-carrier-protein] synthase III protein 1) (Beta-ketoacyl-ACP synthase III 1) (KAS III 1) (bFabH1) | *fabHA* | 3.46 | 0.0001 |
| CAB13463.1 | Malonyl CoA-acyl carrier protein transacylase (MCT) (EC 2.3.1.39) | *fabD* | 0.70 | 0.0394 |
| CAB13461.1 | Transcription factor FapR (Fatty acid and phospholipid biosynthesis regulator) | *fapR* | 6.70 | 0.0001 |
| CAB13464.1 | 3-oxoacyl-[acyl-carrier-protein] reductase FabG (EC 1.1.1.100) (3-ketoacyl-acyl carrier protein reductase) (Beta-Ketoacyl-acyl carrier protein reductase) (Beta-ketoacyl-ACP reductase) | *fabG* | 2.65 | 0.0002 |
| CAB15654.2 | 3-hydroxyacyl-[acyl-carrier-protein] dehydratase FabZ (EC 4.2.1.59) ((3R)-hydroxymyristoyl-[acyl-carrier-protein] dehydratase) ((3R)-hydroxymyristoyl-ACP dehydrase) (Beta-hydroxyacyl-ACP dehydratase) | *fabZ* | 1.26 | 0.0184 |
| CAB12211.2 | Uncharacterized protein YcsD | *ycsD* | 3.21 | 0.1199 |
| CAB12693.1 | Enoyl-[acyl-carrier-protein] reductase [NADPH] FabL (ENR) (EC 1.3.1.104) (Enoyl-acyl carrier protein reductase III) (NADPH-dependent enoyl-ACP reductase) | *fabL* | 0.13 | 0.5088 |
| CAB13029.2 | Enoyl-[acyl-carrier-protein] reductase [NADH] FabI (ENR) (EC 1.3.1.9) (Cold shock-induced protein 15) (CSI15) (NADH-dependent enoyl-ACP reductase) (Vegetative protein 241) (VEG241) | *fabI* | 2.15 | 0.0003 |
| CAB13462.1 | Phosphate acyltransferase (EC 2.3.1.n2) (Acyl-ACP phosphotransacylase) (Acyl-[acyl-carrier-protein]--phosphate acyltransferase) (Phosphate-acyl-ACP acyltransferase) | *plsX* | 3.44 | 0.0000 |
| CAB13465.1 | Acyl carrier protein (ACP) | *acpA* | 2.25 | 0.0010 |
| CAB14199.1 | Glycerol-3-phosphate dehydrogenase [NAD(P)+] (EC 1.1.1.94) (NAD(P)H-dependent dihydroxyacetone-phosphate reductase) (NAD(P)H-dependent glycerol-3-phosphate dehydrogenase) | *gpsA* | 0.24 | 0.0031 |
| CAB13690.1 | Glycerol-3-phosphate acyltransferase (Acyl-PO4 G3P acyltransferase) (Acyl-phosphate--glycerol-3-phosphate acyltransferase) (G3P acyltransferase) (GPAT) (EC 2.3.1.n3) (Lysophosphatidic acid synthase) (LPA synthase) | *plsY* | 3.99 | 0.0005 |
| CAB12793.1 | 1-acyl-sn-glycerol-3-phosphate acyltransferase (1-AGP acyltransferase) (1-AGPAT) (1-acyl-G3P acyltransferase) (EC 2.3.1.n4) (Lysophosphatidic acid acyltransferase) (LPAAT) (Phosphatidic acid synthase) (PA synthase) | *plsC* | 1.59 | 0.0107 |
|  |  |  |  |  |
| **Figure 4** |  |  |  |  |
| CAB14816.1 | Long-chain-fatty-acid--CoA ligase (EC 6.2.1.3) (Long-chain acyl-CoA synthetase) | *lcfA* | 1.81 | 0.1405 |
| CAB15745.1 | Acyl-CoA dehydrogenase (EC 1.3.99.-) | *acdA* | 2.83 | 0.0568 |
| CAB15271.1 | Probable acyl-CoA dehydrogenase (EC 1.3.99.-) | *fadE* | 0.60 | 0.0001 |
| CAB14812.1 | Electron transfer flavoprotein subunit alpha (Alpha-ETF) (Electron transfer flavoprotein large subunit) (ETFLS) | *etfA* | 3.44 | 0.0039 |
| CAB14813.1 | Electron transfer flavoprotein subunit beta (Beta-ETF) (Electron transfer flavoprotein small subunit) (ETFSS) | *etfB* | 1.18 | 0.0151 |
| CAB14814.1 | Probable enoyl-CoA hydratase (EC 4.2.1.17) | *fadB* | 1.97 | 0.0052 |
| CAB13279.1 | Probable 2,4-dienoyl-CoA reductase (EC 1.3.1.34) | *fadH* | 1.68 | 0.4650 |
|  |  |  |  |  |
| **Figure 6** |  |  |  |  |
| CAB14110.1 | Processive diacylglycerol beta-glucosyltransferase (EC 2.4.1.315) (Beta-diglucosyldiacylglycerol synthase) (Beta-DGS) (DGlcDAG synthase) (Glc2-DAG synthase) (Beta-gentiobiosyldiacylglycerol synthase) (Beta-monoglucosyldiacylglycerol synthase) (Beta-MGS) (MGlcDAG synthase) (Beta-triglucosyldiacylglycerol synthase) (TGlcDAG synthase) (Diglucosyl diacylglycerol synthase (1,6-linking)) (Glucosyl-beta-1,6-glucosyldiacylglycerol synthase) (UDP glucosyltransferase) (UDP-glucose:1,2-diacylglycerol-3-beta-D-glucosyltransferase) | *ugtP* | -1.70 | 0.0109 |
| CAB12545.2 | Lipoteichoic acid synthase 1 [Cleaved into: Glycerol phosphate lipoteichoic acid synthase 1 (LTA synthase 1) (EC 2.7.8.-) (Polyglycerol phosphate synthase 1); Processed glycerol phosphate lipoteichoic acid synthase 1] | *ltaSA* | -0.96 | 0.1281 |
| CAB14415.2 | Lipoteichoic acid synthase-like YqgS [Cleaved into: Uncharacterized protein YqgS; Processed uncharacterized protein YqgS] | *ltaSC* | -1.78 | 0.0023 |
| CAB13565.2 | CDP-diacylglycerol--glycerol-3-phosphate 3-phosphatidyltransferase (EC 2.7.8.5) (Phosphatidylglycerophosphate synthase) (PGP synthase) | *pgsA* | 3.01 | 0.1168 |
| CAB14101.1 | Uncharacterized protein YpjQ | *ypjQ* | 1.76 | 0.0196 |
| CAB12023.1 | Phosphatidylserine decarboxylase proenzyme (EC 4.1.1.65) [Cleaved into: Phosphatidylserine decarboxylase alpha chain; Phosphatidylserine decarboxylase beta chain] | *psd* | -0.84 | 0.4238 |
|  |  |  |  |  |
| **Figure 7** |  |  |  |  |
| CAB14358.1 | 1-deoxy-D-xylulose-5-phosphate synthase (EC 2.2.1.7) (1-deoxyxylulose-5-phosphate synthase) (DXP synthase) (DXPS) | *dxs* | -2.01 | 0.0042 |
| CAB11866.1 | 2-C-methyl-D-erythritol 4-phosphate cytidylyltransferase (EC 2.7.7.60) (4-diphosphocytidyl-2C-methyl-D-erythritol synthase) (MEP cytidylyltransferase) (MCT) | *ispD* | -1.33 | 0.2550 |
| CAB11822.1 | 4-diphosphocytidyl-2-C-methyl-D-erythritol kinase (CMK) (EC 2.7.1.148) (4-(cytidine-5'-diphospho)-2-C-methyl-D-erythritol kinase) | *ispE* | -0.46 | 0.4565 |
| CAB11867.1 | 2-C-methyl-D-erythritol 2,4-cyclodiphosphate synthase (MECDP-synthase) (MECPP-synthase) (MECPS) (EC 4.6.1.12) | *ispF* | -0.03 | 0.9490 |
| CAB14437.1 | 4-hydroxy-3-methylbut-2-en-1-yl diphosphate synthase (flavodoxin) (EC 1.17.7.3) (1-hydroxy-2-methyl-2-(E)-butenyl 4-diphosphate synthase) | *ispG* | -1.02 | 0.0032 |
| CAB14446.2 | 4-hydroxy-3-methylbut-2-enyl diphosphate reductase (HMBPP reductase) (EC 1.17.7.4) | *ispH* | -1.03 | 0.0019 |
| CAB14359.2 | Farnesyl diphosphate synthase (FPP synthase) (EC 2.5.1.10) ((2E,6E)-farnesyl diphosphate synthase) (Geranyltranstransferase) | *ispA* | -2.70 | 0.1575 |
| CAB13526.1 | Isoprenyl transferase (EC 2.5.1.-) | *uppS* | 0.68 | 0.0097 |
| CAB15801.1 | Prephenate decarboxylase (EC 4.1.1.100) (Bacilysin biosynthesis protein BacA) (Non-aromatizing prephenate decarboxylase) | *bacA* | 1.24 | 0.0008 |
| CAB13395.2 | UDP-N-acetylglucosamine--N-acetylmuramyl-(pentapeptide) pyrophosphoryl-undecaprenol N-acetylglucosamine transferase (EC 2.4.1.227) (Undecaprenyl-PP-MurNAc-pentapeptide-UDPGlcNAc GlcNAc transferase) | *murG* | -3.05 | 0.0004 |
